# Identification of a Novel Regulatory Gene, *trmE,* that Orchestrates *Salmonella* Flagellar Synthesis and Virulence

**DOI:** 10.3390/microorganisms13071455

**Published:** 2025-06-23

**Authors:** Haoyu Geng, Linyan Luo, Jian Zhang, Jingying Gao, Shizhong Geng, Paul Barrow

**Affiliations:** 1 Key Laboratory of Prevention and Control of Biological Hazard Factors (Animal Origin) for Agrifood Safety and Quality, Ministry of Agriculture of China, Yangzhou University, Yangzhou 225009, China; g020130@163.com (H.G.); luolyd163yx@163.com (L.L.); zj18705275313@163.com (J.Z.); 15839869329@163.com (J.G.); 2 Jiangsu Key Laboratory of Zoonosis, Yangzhou University, Yangzhou 225009, China; 3 Jiangsu Co-Innovation Center for Prevention and Control of Important Animal Infectious Diseases and Zoonoses, Yangzhou University, Yangzhou 225009, China; 4 Joint International Research Laboratory of Agriculture and Agri-product Safety of the Ministry of Education, Yangzhou University, Yangzhou 225009, China; 5 School of Veterinary Medicine, University of Surrey, Daphne Jackson Road, Guildford GU2 7AL, UK; paul.barrow@surrey.ac.uk

**Keywords:** *Salmonella* Enteritidis, *trmE*, flagella, Tn5 transposon, virulence

## Abstract

It is well established that flagella play a critical role in bacterial motility and virulence, and the genes associated with flagellar synthesis and regulation have been extensively characterized. In this study, we identified the *trmE* gene as a novel modulator of flagellar synthesis in *Salmonella* Enteritidis. A transposon (Tn5) mutant library of *Salmonella* Enteritidis (SE) was constructed through bacterial conjugation, followed by screening for motility-deficient mutants. Among 1321 mutants screened, C50041*trmE*::Tn5 exhibited reduced motility. To validate this phenotype, we constructed C50041Δ*trmE* mutants and complementary strains C50041Δ*trmE*::*trmE*. Compared to parental strain SE(C50041), C50041Δ*trmE* displayed significantly lower mRNA levels of flagellar synthesis-related genes as determined via quantitative real-time PCR and the few visible flagella observed via transmission electron microscopy (TEM). Function studies assessing virulence also showed results that matched this phenotype; specifically, C50041Δ*trmE* demonstrated decreased adhesion and invasion capabilities towards macrophages. Furthermore, C50041Δ*trmE* induced impaired apoptosis and pyroptosis in macrophages, while exhibiting reduced mortality in BALB/c mice along with diminished mRNA levels of pro-inflammatory cellular factors within murine spleen. This study provides compelling evidence that the *trmE* gene in *Salmonella* Enteritidis is involved in flagellar synthesis.

## 1. Instruction

*Salmonella* spp. are critical zoonotic pathogens that are predominantly transmitted via the fecal–oral route in animals and humans [1]. They can survive in macrophages in vivo after being engulfed, and subsequently escape to cause systemic infections [2,3]. This process establishes the *Salmonella* infection cycle, triggering a robust inflammatory response leading to severe disease.

In *Salmonella’s* pathogenesis, flagella play a crucial role in various processes, such as biofilm formation [4], inflammation induction [5], growth chemotaxis [6], and intracellular escape [7], which contribute to their survival in the host environment. The structural components of flagella include three main elements: the basal body, hook, and filament [8]. Many regulators and chaperones are known to take part in its synthesis [9]; almost 50 genes have been manipulated by more than 17 operons to synthesize flagella in *Salmonella typhimurium* [10] to date. Protein synthesis is a highly complex process, and in addition to those mentioned above, there are many constraints, such as DNA transcription, mRNA modification, and protein translation. We believe that a few regulators—beyond the controlling genes of the flagella themselves—remain undiscovered, but identifying these regulators will necessitate specialized methods.

*Salmonella enterica* may show flagellar phase variation; its flagellin filament protein has two forms, either type B or C. This switching is achieved through the stochastic inversion of a promoter that produces both type B flagellin (FljB) and an inhibitor (FljA) of type C flagellin formation. When the fljB-fljA operon is expressed, only type B flagella are produced; when the operon is not transcribed, the gene for type C flagellin (FliC) is released from inhibition and forms type C flagella [11].

In addition, *Salmonella* flagella do not always exist during the pathogen’s life cycle. When *Salmonella* enters host cells, flagella synthesis is quickly turned off to escape the host immune system. Two EAL-like proteins in Salmonella, STM1344 and STM1697, are involved in the cooperative regulation of flagellar synthesis [12].

Although most genes have been identified in different bacteria, flagellar synthesis systems of *Escherichia coli* and *Salmonella enterica* exhibit a significant level of genetic and functional synteny, although they may not function in precisely the same manner [13].

We believe that some flagella-synthesized gene(s) remain unidentified. Transposon mutagenesis is a powerful reverse genetic technique that can be used to find such function-related genes based on the principle of randomly inserting transposons into the bacterial genome [14]. We have discovered many function-related genes in *Salmonella* using this technique. This method has the special advantage of offering a great number of mutants in the transposon mutant library, and a lot of target mutants can be quickly screened out one by one so many function-related genes can be identified.

This study employed this advanced technique to find genes regulating flagellar synthesis. One SE(C50041) mutant library was constructed with a plasmid pUT miniTn5, and mutants with weak bacterial motility were screened out on semi-solid LB media. The selected mutants underwent whole genome-sequencing to identify target genes using NCBI BLAST using transposon Tn5-flanking sequences. Flagella were observed via TEM to further confirm motility phenotype. Subsequently, function analysis was conducted to further confirm bacterial attenuated virulence in both cellular and murine models.

## 2. Materials and Methods

### 2.1. Bacterial Strains, Plasmids, and Cells

The bacterial strains, plasmids, and cells used in this study are listed in Table A1. The defined bacterial mutants were constructed according to the method of genome genetic modification [15].

### 2.2. Mice and Animal Ethics

Specific pathogen-free (SPF) female BALB/c mice (8 week; 20 ± 2 g) were obtained from the Comparative Medical Center of Yangzhou University (Yangzhou, China). Animal experiments were approved by the Animal Welfare and Ethics Committees of Yangzhou University (SYXK[Su] 2023-0089) and were conducted in accordance with the guidelines of the Yangzhou University Institutional Animal Care and Use Committee (IACUC).

### 2.3. Construction of SE Transposon Mutant Library

A Tn5 mutant library was constructed by conjugating donor *E. coli* χ7213 (Tn5), which is growth-dependent to DAP (diaminopimelic acid) and resistant to chloramphenicol and kanamycin, with recipient SE C50041, which is sensitive to kanamycin. A suicide vector pUT with mini-Tn5 transposon in *E. coli* χ7213, in which a kanamycin-resistant gene as a transposon can be inserted into bacterial chromosome through transposase. Each transconjugant was isolated on LB agar containing 50 µg/mL chloramphenicol and 100 µg/mL kanamycin and no DAP [16], because *E. coli* χ7213 (Tn5) cannot grow in LB media without DAP and SE C50041 cannot grow in LB media with kanamycin, and so only transconjugant as a mutant can grow in LB media with kanamycin and without DAP.

### 2.4. Motility-Deficient Mutant Screening and Identification using Semi-Solid Plate and U-Tube

Each mutant was inoculated into LB liquid medium, the concentration was adjusted to OD600 = 1 using PBS, and then 10 μL of bacterial suspension was pipetted onto the center of the semi-solid plate containing 0.5% agar. After the bacterial suspension was dried, the plate was incubated in a constant-temperature incubator for 20 h at 37 °C. *Salmonella* motility can be statistically analyzed using the diameter of the bacterial halo [16].

Subsequently, the mutant with weak motility was evaluated again using a U-tube assay. A sterile needle with a freshly cultured single colony of SE mutant was punctured into semi-solid LB medium from one side of a U-tube. The U-tube was placed in a constant-temperature incubator for 20 h, and bacterial growth was observed on the LB surface from another side of the U-tube.

### 2.5. Gene Identification of Sequence Flanking Tn5 Inserted in Bacterial Genome

The confirmed mutant with weak motility underwent bacterial whole-genome sequencing, where the bacterial genome was sequenced on an Illumina HiSeq platform (Annaroad Gene Technology, Beijing, China) and assembled using MicrobeTrakr Plus 0.9.1. Tn5 (kanamycin-resistant gene) and its flanking sequence were found, and a Tn5-inserted target gene was identified through homology searches using the public databases BLASTn and BLASTx at http://www.ncbi.nlm.nih.gov (accessed on 22 June 2022).

### 2.6. Construction of C50041∆trmE and Its Complemented Strain C50041∆ trmE::trmE

According to the protocol based on pGMB152 suicide plasmid [17], C50041∆*trmE* was constructed with the chloramphenicol resistance gene replacing the *trmE* gene, and the complemented strain C50041∆*trmE*::*trmE* was generated with pBR322-*trm*E [18].

### 2.7. Biological Characteristics of SEΔtrmE

#### 2.7.1. Growth and Biochemical Characteristics

C50041Δ*trmE* and SE(C50041) were inoculated into 4 mL of LB liquid media and incubated at 37 °C with continuous shaking (180 rpm) for 8–10 h. Bacterial cultures were adjusted to an OD600 of approximately 0.05 using LB medium. The adjusted suspensions were then transferred to a 96-well plate (200 μL per well), with three replicate wells per strain. Measurements were performed using the Spark multimode microplate reader (Switzerland) with the incubation conditions set to 37 °C and 180 rpm shaking. Bacterial growth was monitored once per 30 min for 12 h through the systematic measurement of OD600 values, and the recorded data were subsequently used for the growth curve.

API 20E biochemical identification strips (BioMérieux, Craponne, France) were utilized to assess the biochemical characteristics of C50041Δ*trmE* and SE(C50041). A fresh single colony was picked using an inoculating loop to a 0.85% saline solution to be cultured to OD600 = 0.6–0.8. According to the manufacturer’s protocol, bacterial suspension was added to the reaction wells of the test strip, and the reaction wells of URE, LDC, ADH, H_2_S, and ODC needed be sealed with liquid paraffin. Then, the test strip was placed on honeycomb in a sealed metal box, with 5 mL of distilled water added to fill its recesses for a humid environment. After incubation at 30 °C for 24 h, chromogenic reagents were added, and color changes were observed [19].

#### 2.7.2. Antimicrobial Susceptibly Test

Antimicrobial susceptibility testing was performed using the CLSI disk diffusion method. Bacterial colonies from MH agar were suspended in 4.5% NaCl to achieve the 0.5 McFarland standard. The suspension was swabbed onto MH plates using three-directional streaking. In total, 12 antibiotic disks (Oxoid) were aseptically applied, and the plates were incubated at 37 °C (16–18 h). Inhibition zones were measured using digital calipers and interpreted per CLSI Enterobacteriaceae guidelines. Quality control strains (*E. coli* ATCC 25922, *K. pneumoniae* ATCC 700603) validated each assay [20].

### 2.8. mRNA Level of Flagellar Synthesis-Related Gene and Flagella Observation via TEM

#### 2.8.1. mRNA Level of Flagellar Synthesis-Related Gene via qRT-PCR

C50041Δ*trmE* and SE(C50041) were cultured for almost 10 h, bacterial RNA was extracted using an RNA extraction kit (TianGen Biotech (Beijing) Co., Ltd., Beijing, China), and the concentration and purity of the RNA were assessed using a micro-spectrophotometer. According to the instructions in the PrimeScript™ RT reagent kit (Takara Company, Beijing, China), cDNA was synthesized, and the mRNA level of flagellar synthesis-related genes was detected via qRT-PCR based on the cDNA, with the bacterial 16S rRNA gene used as an internal reference gene [21].

#### 2.8.2. Flagella Observation via TEM

A fresh single bacterial colony was cultured statically for 10 h, suspended with PBS, and negatively stained with 0.1% phosphotungstic acid solution for 1 min; flagella were observed via TEM [16].

### 2.9. In Vitro and In Vivo Assay for Virulence Analysis

#### 2.9.1. Cell Adhesion, Invasion, and Intracellular Proliferation

Cell Adhesion. Murine macrophages RAW264.7 and J774A.1 were cultured in complete DMEM medium and maintained in a 37 °C incubator with 5% CO_2_. After viability assessment, they were digested with pre-warmed trypsin and incubated at 37 °C for 1 min (RAW264.7) or 5 min (J774A.1) and then seeded at a density of 2.0 × 10^5^ cells per well in a 24-well plate for 12 h. The medium was discarded and replaced with fresh DMEM, and 100 μL of SE suspension at a multiplicity of infection (MOI) of 100:1 was then added. After a 30 min adhesion, the medium was discarded again, and the cell wells were washed twice with sterile PBS. Subsequently, 1 mL of 0.2% Triton X-100 was added to lyse the cells at 37 °C for 10 min. Diluted bacteria were coated on the LB plate and counted, and the adhesion rate of the mutant relative to SE(C50041) was calculated; three replicates were carried out for each mutant.

Cell Invasion. The preliminary steps were identical to those in the adhesion experiment. After 30 min for adhesion, the medium was removed, the cell wells were washed with sterile PBS, and 1 mL of fresh DMEM (Dulbecco’s Modified Eagle’s Medium) was added with 100 μg/mL gentamicin. Then, 1 h later, the medium was discarded, the cell wells were washed twice with sterile PBS and lysed with 0.2% Triton X-100, diluted bacteria were coated on the LB plate, bacterial colonies were counted, and the invasion rate of the mutants relative to SE(C50041) was calculated, with three replicates carried out for each mutant.

Intracellular Proliferation. The preliminary steps were consistent with those in the invasion experiment. After incubating the cells in DMEM containing 100 μg/mL gentamicin for one hour, the cells were washed twice with sterile PBS and then cultured in 1 mL of DMEM containing 10 μg/mL gentamicin, and then the cells at 1 h, 5 h, 10 h, and 20 h post cell culture were lysed to count the number of intracellular bacteria and plot the proliferation curve, with three replicates carried out for each mutant.

#### 2.9.2. LDH for Cytotoxicity

The cytotoxicity of *trmE* mutants was evaluated using the LDH (Lactate dehydrogenase) release from *Salmonella*-infected RAW264.7 macrophages. Cell culture and bacterial infection were performed as described above. DMEM with 10 µg/mL gentamicin was added for 6 h, and the LDH level in the cell media was detected using an LDH cytotoxicity detection kit (Beyotime, Nantong, China), with three replicates.

#### 2.9.3. Apoptosis Based on Dual Staining with FITC and PI

After the RAW264.7 cells were infected with *Salmonella* strains, the degree of macrophages apoptosis was analyzed via flow cytometry using the Annexin V-FITC Kit (Miltenyi, Bergisch Gladbach, Germany) for labeling cells. After RAW264.7 cells were infected with *Salmonella* for 3 h, they were collected and counted. Following the protocol in the Annexin V-FITC Kit, the cells were stained with FITC and PI and then analyzed via flow cytometry to calculate the rate of macrophage apoptosis [22].

#### 2.9.4. Pyroptosis Based on Caspase-1 Protein

After RAW264.7 cells were infected with *Salmonella* strains, the degree of macrophages pyroptosis was assessed by measuring the protein level of Caspase-1. *Salmonella* strains were cultured in liquid LB for 12 h. Following a wash with sterile PBS, the optical density was adjusted to OD600 = 1 for further use. J774A.1 cells were digested with trypsin and seeded at a density of 5.0×10^5^ cells per 500 μL in a 12-well plate for culturing 12 h. After the cells were pre-stimulated with 1 mg/mL LPS for 5 h, the culture media were then replaced with fresh OPTI-MEM media, and bacteria were added at MOI = 100:1. After 30 min for adhesion, the media were replaced with OPTI-MEM containing 50 µg/mL gentamicin for an additional 3 h. Cell media were collected and cells were lysed using 500 µL of lysis buffer. After protein extraction and concentration from the collected cell media and cell lysate, concentrated products were resuspended in 40 μL of 1 × SDS sample buffer and incubated at 95 °C for 10 min for full dissolution. Finally, Caspase-1 protein in the samples was analyzed using Western blot [23].

#### 2.9.5. LD_50_ in Mice

C50041Δ*trmE*, C50041*trmE*::Tn5, and SE(C50041) were inoculated into 4 mL of LB liquid medium and incubated overnight at 37 °C with shaking at 180 rpm, and then bacterial cultures were washed with sterile PBS and adjusted to an optical density of OD600 = 1. Then, 10-fold serial dilutions from 1.0 × 10^4^ CFU to 1.0 × 10^8^ CFU were administered orally to 8-week-old female BALB/c mice per mouse (*n* = 5/group), while a PBS group was established as a blank control [24]. The mice were monitored for two weeks, during which their survival was recorded and LD_50_ was calculated. The logarithmic value of the maximum dose group, the logarithmic ratio of adjacent high and low doses, and the total mortality rate across all groups were denoted as Xm, i, and ΣP, respectively. Then, the formula for calculating LD_50_ is LD_50_ = log − 1[Xm − i(ΣP − 0.5)].

#### 2.9.6. Persistence In Vivo

After oral administration, mice (*n* = 3 per group) were euthanized at days 1, 4, and 7 post *Salmonella* infection. Spleens and livers were aseptically harvested, weighed, and then homogenized in 1 mL of sterile PBS using a homogenizer at 4500 rpm for 15 s, two times. The homogenate was serially diluted, and three appropriate dilutions were coated on an XLT4 plate for 12 h incubation until black colonies emerged. Bacterial counts were performed on the plates to analyze *Salmonella* persistence in vivo [16].

#### 2.9.7. Lesions and mRNA Level of Inflammatory Cytokine in Murine Spleen

After the *Salmonella* strains orally infected the mice, murine spleens were collected on days 1, 4, and 7. One tissue part was fixed in 10% formalin and processed into paraffin sections for lesion analysis, while another part was homogenized using a homogenizer to extract total mRNA, and its cDNA was analyzed via qPCR, as described in Section 2.8.1. The relative mRNA levels of inflammatory cytokines were compared to those of murine GAPDH, which served as an internal reference gene. The primers used are listed in Table A2 and Table A3.

### 2.10. Statistical Analysis

The data regarding bacterial CFUs, mice survival, and motility analysis were analyzed using GraphPad Prism 10 (GraphPad Software, LaJolla, CA, USA). An analysis of variance (ANOVA) was performed by comparing mutant groups to the SE(C50041) control and blank control. All results are expressed as the mean ± SEM. Statistical significance was assigned at *p* < 0.05 (*), <0.01 (**), <0.001 (***), and <0.0001 (****) based on Student’s *t*-test.

## 3. Results

### 3.1. Phenotype Exhibited trmE::Tn5 Mutant Without Motility

A total of 1321 mutants were screened from the Tn5 mutant library of SE(C50041), which was estimated to contain more than 20,000 mutants, and 11 mutants showed motility deficiencies. Through BLAST analysis based on the bacterial whole-genome sequence, a Tn5-inserted gene was identified through homology searches of the Tn5 flanking sequence. Eight genes were identified, as shown in Table 1: *trmE* gene (tRNA modification GTPase TrmE), *fliD* (two mutants), *fliP* and *fliA* genes (flagella synthesis), *rfbK* and *rfaL* (two mutants) *genes* (lipopolysaccharide synthesis), *csrD gene* (two mutants) (global regulation). Although most Tn5-inserted genes were annotated as components or regulators associated with the flagella, the *trmE* gene was focused on because it has not been previously reported for bacterial motility and flagella synthesis.

### 3.2. SE∆trmE Reconfirmed This Phenotype Without Motility

**Figure 1:**
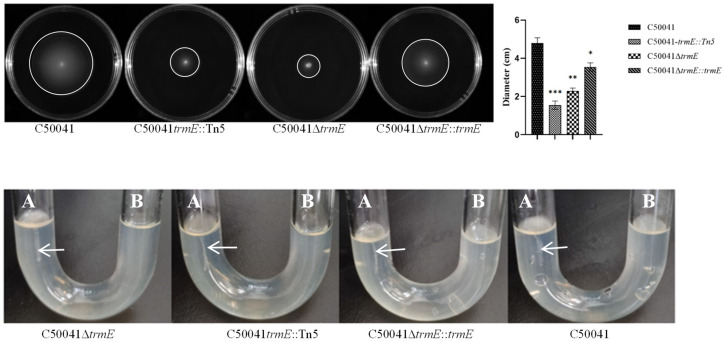
Motility analysis of SE *trmE* mutants using semi-solid plate and U-tube. Note: A is the puncture end, and B is the observation end. *p* < 0.05 (*), <0.01 (**), <0.001 (***).

To confirm the phenotype, C50041∆*trmE* and C50041∆*trmE*::*trmE* were constructed and compared to their motility utilizing the semi-solid LB plate and U-tube. Compared to C50041*trmE*::Tn5, C50041∆*trmE* exhibited the same phenotype without motility on a semi-solid plate, while C50041∆*trmE*::*trmE* displayed a phenotype with strong motility, which was similar to that of SE (C50041) (Figure 1). The diameter of the SEΔ*trmE* halo was measured at 23 ± 1 mm, while that of C50041*trmE*::Tn5 was 15 ± 1 mm and that of C50041∆*trmE*::*trmE* was 36 ± 2 mm. As a control, that of the SE(C50041) halo was up to 48 ± 2 mm. These results indicate that SEΔ*trmE* motility was significantly weaker than that of SE(C50041) (*p* < 0.05).

This was assessed again using U-tube assays. The needles were punctured into LB media from the A side of the U-tube, and bacterial growth was observed on the LB surface from the B side. C50041Δ*trmE* and C50041*trmE*::Tn5 showed no bacterial growth, while C50041∆*trmE*::*trmE* and SE(C50041) grew on the surface at B side of the U-tube, which indicates that C50041Δ*trmE* had no motility (Figure 1) by *trmE* loss.

### 3.3. Biological Characteristics of C50041ΔtrmE Without Flagella

#### 3.3.1. No Change in Biochemical Characteristics

After biochemical identification card API 20E was employed, the biochemical characteristics of C50041Δ*trmE* were confirmed to be consistent with those of SE(C50041), and C50041∆*trmE*::*trmE*, ONPG, ADH), LDC, CIT, URE, TDA, IND, VP, GEL), INO, SAC, MEL, and AMY were tested to be negative (−), while ODC, H_2_S, GLU, MAN, SOR, RHA, and ARA were detected to be positive (+). This indicates that the *trmE* gene did not affect the biochemical properties of SE(C50041).

#### 3.3.2. No Change in Antibiotic Resistance

Antimicrobial susceptibility in SE(C50041), C50041Δ*trmE*, *and* C50041∆*trmE*::*trmE* was tested using 12 antibiotic disks (Oxoid, Basingstoke, UK) with the following specifications: kanamycin (KAN, 20 μg), ciprofloxacin (CIP, 5 μg), meropenem (MEM, 10 μg), cefotaxime (CTX, 5 μg), ampicillin (AMP, 10 μg), chloramphenicol (CHL, 30 μg), tetracycline (TET, 30 μg), nalidixic acid (NAL, 30 μg), florfenicol (FFC, 30 μg), streptomycin (STR, 10 μg), gentamicin (GEN, 10 μg), and trimethoprim-sulfamethoxazole (SXT, 10 μg). This demonstrated that C50041Δ*trmE* did not alter bacterial antibiotic resistance.

#### 3.3.3. No Change in Bacterial Growth Speed

**Figure 2:**
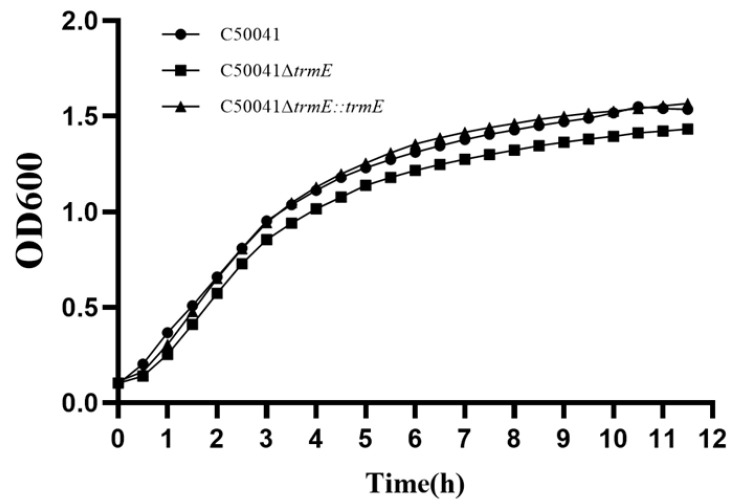
Growth curves of *Salmonella* strains.

The growth speed of C50041Δ*trmE* was analyzed by measuring its absorbance at 600 nm for the growth curve, indicating that the growth rate of C50041Δ*trmE* showed no significant difference at the same time points from 1 to 12 h compared to SE(C50041) and C50041∆*trmE*::*trmE* (Figure 2).

### 3.4. Low mRNA Level of Flagella Synthesis-Related Genes of C50041ΔtrmE

**Figure 3:**
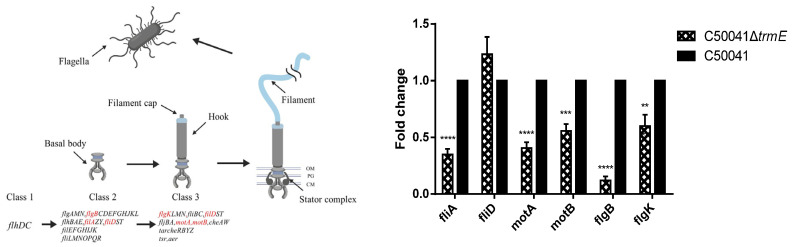
mRNA level of part C50041Δ*trmE* flagella synthesis-related genes and the locations of these genes (marked in red) [25]. *p* < 0.01 (**), <0.001 (***), <0.0001 (****).

The mRNA level of the flagella synthesis-related genes of C50041Δ*trmE* was analyzed via qRT-PCR. C50041Δ*trmE* was indicated to be significantly downregulated in terms of the mRNA level of the flagellar synthesis σ factor *fliA* (*p* < 0.05), flagellar motor proteins *motA* (*p* < 0.05) and *motB* (*p* < 0.05), and flagellar biosynthesis proteins *flgB* (*p* < 0.05) and *flgK* (*p* < 0.05). This evidence demonstrates that *trmE* loss significantly inhibited flagellar synthesis in C50041Δ*trmE* (Figure 3).

**Figure 4:**
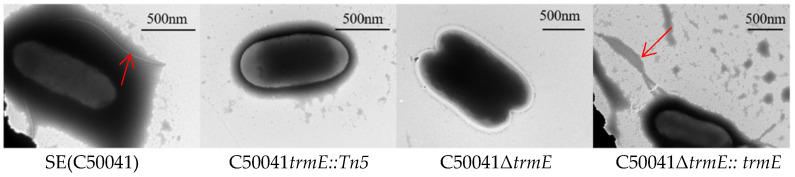
Flagellar observation of *SE trmE* mutants via TEM. Note: The red arrows point to *Salmonella* flagella.

Further, *Salmonella* strains were observed for flagella via TEM: C50041Δ*trmE* exhibited few flagella on the bacterial surface compared to SE(C50041) and C50041Δ*trmE*:: *trmE*, which showed many flagella (Figure 4).

### 3.5. Low-Virulence In Vitro and In Vivo Assay of C50041ΔtrmE

#### 3.5.1. Low Ability to Infect Macrophages

**Figure 5:**
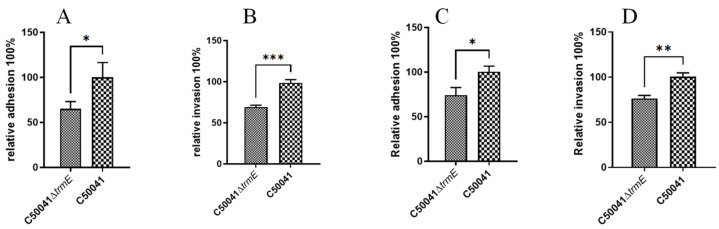
Adhesion and invasion ability of C50041Δ*trmE* to RAW264.7 (**A**,**B**) and J774A.1 (**C**,**D**) cells (MOI = 100). *p* < 0.05 (*), <0.01 (**), <0.001 (***).

RAW264.7 and J774A.1 cells were infected with *Salmonella* strains at MOI = 100:1. Adhesion and invasion abilities were evaluated after 30 min adhesion and then 1 h invasion. C50041Δ*trmE* exhibited a significant decrease in adhesion rates to RAW264.7 (*p* < 0.05) and J774A.1 (*p* < 0.001) and in invasion rates to RAW264.7 (*p* < 0.05) and J774A.1 (*p* < 0.01) compared to SE(C50041) (Figure 5), which suggests that the *trmE* gene significantly affected *Salmonella* adhesion and invasion abilities to RAW264.7 and J774A.1 cells.

**Figure 6:**
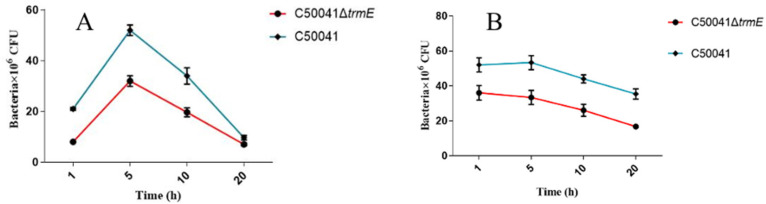
Proliferation ability of C50041Δ*trmE* in RAW264.7 (**A**) and J774A.1 (**B**) cells.

Subsequently, the proliferation rate of C50041Δ*trmE* was assessed by counting the intracellular bacterial numbers at 1, 5, 10, and 20 h post infection using dilution plating. The results demonstrate that the loads of C50041Δ*trmE* were significantly low at every time point compared to those of SE(C50041) because the primary intracellular bacterial numbers were different. However, the change trend was similar, indicating that the *trmE* gene did not affect the proliferation capacity of SE(C50041) (Figure 6).

#### 3.5.2. LDH Assay for Cytotoxicity

**Figure 7:**
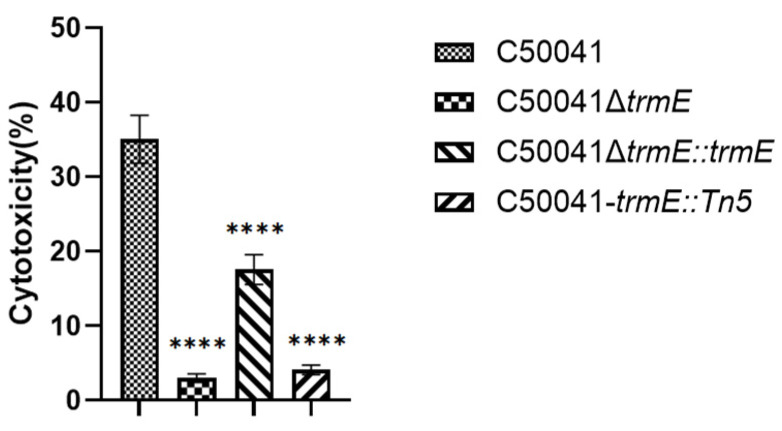
Cytotoxicity of *Salmonella* to RAW264.7 based on LDH assay. *p* <0.0001 (****).

The cytotoxic activity of C50041Δ*trmE* was assessed against RAW264.7 using a lactate dehydrogenase (LDH) release assay (Figure 7). C50041Δ*trmE* could induce a statistically significant reduction in LDH release from RAW264.7 cells (*p* < 0.0001) compared to SE (C50041), which demonstrates that *trmE* loss attenuated SE(C50041) cytotoxicity.

#### 3.5.3. Decreased Ability to Induce Macrophage Apoptosis

**Figure 8:**
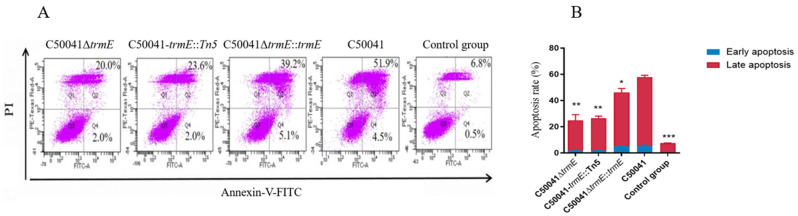
Ability analysis of *Salmonella* to induce macrophage apoptosis. (**A**): Flow cytometry; (**B**): statistical analysis. *p* < 0.05 (*), <0.01 (**), <0.001 (***).

After 3 h of RAW264.7 cells being infected by *Salmonella* strains, the early and late macrophage apoptosis level was assessed via flow cytometry using the Annexin V-FITC Kit. In addition to live cells (Annexin V^−^/PI^−^), the early apoptosis (Annexin V^+^/PI^−^) rate of RAW264.7 cells was low from 2.0% to 5.1%, due to different *Salmonella* strains, but the late apoptosis (Annexin V^+^/PI^+^) rate with C50041Δ*trmE* was 20.0%, which is same as that of C50041*trmE*::Tn5(23.6%) (*p* < 0.05) and significantly lower than that of C50041Δ*trmE*::*trmE* (39.2%) (*p* < 0.05) and SE(C50041) (51.9%) (*p* < 0.01) (Figure 8). This suggests that *trmE* loss significantly reduced the ability of SE-induced macrophage apoptosis.

#### 3.5.4. Decreased Ability to Induce Macrophage Pyroptosis Based on Caspase-1 Level

**Figure 9:**
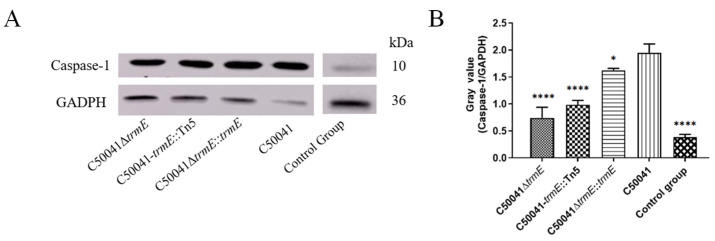
Caspase-1 level during *Salmonella* C50041Δ*trmE* inducing macrophage pyroptosis. (**A**): Western blot analysis; (**B**): gray analysis. Note: The original image was submitted. *p* < 0.05 (*), <0.0001 (****).

Caspase-1 level was assessed in RAW264.7 cells after *Salmonella* infection using Western blot, which reflected *Salmonella*’s ability to induce macrophage pyroptosis. The results indicate that the Caspase-1 level with C50041Δ*trmE* was similar to that of C50041*trmE*::Tn5, significantly reduced compared to that with SE(C50041) (*p* = 0.0016), which demonstrates that *trmE* loss significantly inhibited SE to induce macrophage pyroptosis (Figure 9).

#### 3.5.5. Low Mortality to Mice Based on LD_50_

LD_50_ was determined for C50041Δ*trmE* and SE(C50041). Ten-fold gradient concentrations from 1.0 × 10^4^ CFU to 1.0 × 10^8^ CFU per mouse were orally administered to 8-week-old female BALB/c mice (*n* = 5), which were continuously observed for 2 weeks to record their survival. LD_50_ was calculated using the method described by Kouchi. The LD_50_ of C50041Δ*trmE* increased by over 1500 folds compared to that of SE(C50041). This indicates that *trmE* loss significantly reduced SE virulence.

#### 3.5.6. Low Persistence Ability In Vivo

**Figure 10:**
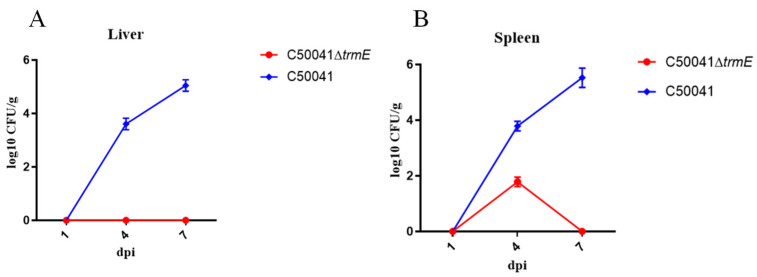
Dynamic analysis of *Salmonella* strains in murine liver (**A**) and spleen (**B**).

According to LD_50_, a high dose of 1.0 × 10^7^ CFU in 100 µL C50041∆*trmE* was orally administered to mice (*n* = 3). The in vivo dynamics showed that no bacteria in the liver and only a few in the spleen were isolated, which shows that *trmE* loss decreased SE persistence ability in vivo (Figure 10).

#### 3.5.7. No Obvious Tissue Lesions and Changed mRNA Level of Pro-Inflammatory Cytokines

**Figure 11:**
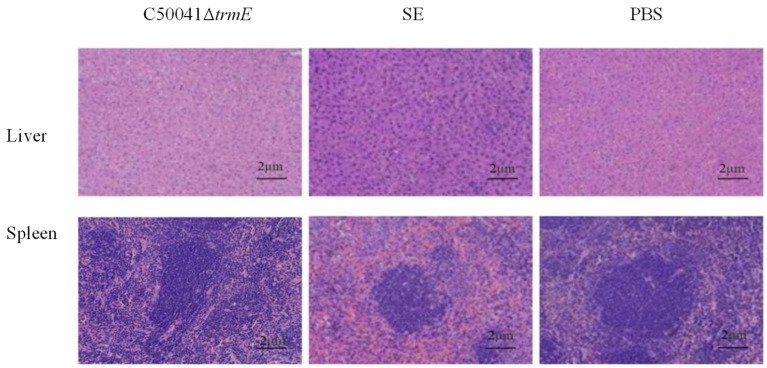
Lesions in murine liver and spleen from *Salmonella* infection (200×). Note: Compared with the C50041Δ*trmE* and PBS group, the SE group exhibits a splenic nodule architecture that is markedly disorganized, exhibiting significant depletion of small lymphocytes within the nodules. Cellular arrangement appears loose, with effacement of the red-white pulp demarcation, accompanied by diffuse inflammatory cell infiltration.

On day 7 after the mice (*n* = 3) were infected with *Salmonella* strains, pathological sections of the spleen and liver were taken and analyzed. No significant lesions occurred in either organ in the C50041Δ*trmE* group, but there were severe lesions with inflammatory cell infiltration in the SE(C50041) group as a control and local necrotic foci. This proves that the *trmE* loss largely reduced *Salmonella* virulence (Figure 11).

**Figure 12:**
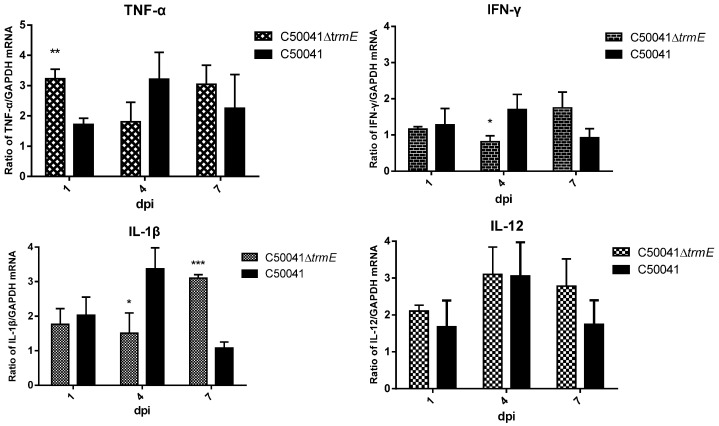
mRNA level of murine cytokines induced by *Salmonella* strains in spleen (using three mice, three replicates per sample, and a *t*-test). *p* < 0.05 (*), <0.01 (**), <0.001 (***).

In addition to tissue lesions, the mRNA of the pro-inflammatory factor also changed. After C50041Δ*trmE* infection, there was a significant increase in the mRNA of TNF-α (*p* < 0.01) on day 1 and IL-1β (*p* < 0.001) on day 7, but the mRNA of IL-1β (*p* < 0.05) and IFN-γ (*p* < 0.05) was significantly reduced on day 4 compared to that of SE (C50041). IL-12 had no difference compared to that of SE(C50041) (Figure 12).

## 4. Discussion

The flagella of *Salmonella* play a crucial role in the bacteria’s bacterial motility and virulence. Utilizing its flagella, *Salmonella* increases the likelihood of being captured by resident macrophages in tissues [26], thereby breaching the blood–intestinal barrier and entering the internal environment. Once engulfed by macrophages, *Salmonella* proliferates within the *Salmonella-containing vacuole* (SCV) and activates the inflammasome through flagellin, leading to the activation of Caspase-1 and the initiation of the classical pyroptosis pathway. This ultimately results in macrophage pyroptosis and contributes to systemic infection [27]. Research on the regulatory genes of flagella holds significant importance for comprehensive understanding of *Salmonella* pathogenicity and virulence.

To identify all genes related to function or phenotype, a mutant library must be constructed and high-throughput screening must be performed. Theoretically, the *Salmonella* ssp. genome has more than 4000 ORFs; a minimum number of mutants equal to or two times greater than the number of ORFs is required for a mutant library to be considered representative. Although high-throughput screening is performed, not all genes related to function or phenotype are screened out in one experiment. In this study, we aimed to find novel gene(s) related to flagella synthesis; however, this is difficult to achieve because most genes have been found. In total, 11 motility-deficient mutants were screened out, and 8 genes were identified because 1 gene was possibly identified in two or more mutants. The other seven genes except *trmE* were confirmed to be involved in flagellar synthesis. A motility-deficient *trmE* mutant was accidentally identified from 1321 mutants in one transposon mutation library. Currently, there is no research reporting that the loss of the *trmE* gene results in the loss of *Salmonella* flagella. A thorough analysis of this mutant based on the motility phenotype and virulence function confirmed that the *trmE* gene regulates flagellar synthesis to reduce bacterial virulence.

The *trmE* gene was inactivated through its insertion with a Tn5 transposon, which resulted in SE(C50041) with weak motility. Thus, we constructed C50041Δ*trmE* and C50041Δ*trmE*::*trmE* to demonstrate their ability to regulate flagellar synthesis to prove their close correlation. At last, a few flagella on the surface of C50041Δ*trmE* were observed via TEM as direct evidence, but their growth speed and biochemical characteristics did not change compared to with SE(C50041). Then, virulence analysis showed that the adhesion and invasion abilities of C50041Δ*trmE* were decreased, as well as the intramacrophage proliferation capacity, cytotoxicity, cell apoptosis and pyroptosis, LD_50_, and tissue lesions.

Studies have indicated that region VIII of the *fliC* gene, which encodes flagellin, serves as a primary site for the induction of cytokines such as TNF-α and IFN-γ in CD^4+^ T cells [28]. Flagella can also stimulate PBMCs to release cytokines such as IL-1β and IL-12 [29]. Thus, we analyzed pathological changes in murine liver and spleen and the mRNA level of inflammatory factors in murine spleens in order to prove that C50041Δ*trmE* successfully infected and induced localized tissue lesions, revealing that the absence of the *trmE* gene significantly affected the degree of inflammatory response in mice. However, the mRNA level of inflammatory factors in murine spleens did not show regularity. The absence of flagella does not lead to a comprehensive reduction in the expression of pro-inflammatory cytokines. For example, the levels of these pro-inflammatory cytokines were consistently elevated in the C50041Δ*trmE* group compared to the wild-type control group on day 7 post Salmonella infection, and the mRNA level of IL-12 showed no change on days 1, 4, or 7, which did not match theoretical expectations, suggesting the potential existence of other compensatory mechanisms that need further investigation.

The *trmE* gene’s involvement in bacterial virulence has been studied. During protein translation, the wobble effect occurs, and the third (5′) base of the anticodon can typically pair with either member of the purine or pyrimidine pair in the codon. The *trmE* gene has been studied to decode TrmE to modify U34. It is also referred to as *mnmE* or *thdF*, and it spans 1364 base pairs. The *trmE* gene encodes the tRNA-modifying GTPase TrmE, which introduces a carboxymethyl-amino-methyl (cmnm) modification at the swing position (U34) of selected tRNAs, leading to the creation of tRNA-cmnm5s2U34 [30] for translational misreading [31]. The TrmE protein exhibits a tripartite structural domain and is highly conserved across eukaryotic and bacterial species; it functions as a molecular switch, adopting various conformations based on GTP or GDP binding. To date, researchers have identified over 20 homologs of TrmE in both prokaryotes and eukaryotes. Additionally, *trmE* genes may play a role in bacterial survival under low-temperature conditions. Singh et al. demonstrated that low temperatures induce the *trmE* gene and its promoter, noting that the Pseudomonas syringae CSM1 mutant, which lacks a functional *trmE* gene, exhibits cold sensitivity [32,33]. Gong investigated the involvement of the *trmE* gene in *E. coli* acid tolerance, revealing that this effect depends on glutamate and requires the activation of the *gadE* gene [34]. Currently, there are no documented studies indicating that no expression of the *trmE* gene leads to the loss of *Salmonella* flagella.

Although we have confirmed that the *trmE* gene is positively correlated with flagellar synthesis, we also found one question asking why it significantly affects flagella synthesis: since TrmE affects the function of tRNA, it should have a global impact, but no significant effect was observed on its growth speed, biochemical characteristics, or antibiotic resistance. This bias and its mechanism will be elucidated in the future to further explore the mysteries of bacterial life.

## 5. Conclusions

In conclusion, our study provides strong evidence, from the phenotype and function levels, that *trmE* is a novel gene involved in the regulation of flagella synthesis and bacterial virulence.

## Figures and Tables

**Table 1:** Information regarding the insertion of Tn5 into genes.

Identified Gene	Number of Mutant	Gene Function	Similarity
*trmE*	1	tRNA modification GTPase TrmE	100%
*fliD*	2	synthesis of flagellum	100%
*fliP*	1	synthesis of flagellum	100%
*rfbK*	1	synthesis of lipopolysaccharides (LPS)	100%
*fliA*	1	flagellar biosynthesis sigma factor	100%
*cpsG*	1	synthesis of lipopolysaccharides (LPS)	100%
*rfaL*	2	synthesis of lipopolysaccharides (LPS)	100%
*csrD*	2	Global regulation	100%

## Data Availability

Data are contained within the article.

## Appendix A

**Table A1:** Bacterial strains, plasmids, and cells used in this study.

Strains or Plasmids	Characteristic	Reference
Strains		
SE C50041	Wild-type *Salmonella* Enteritidis	Lab collection
C50041-*trmE*::Tn5	C50041 with Tn5 inserted in *trmE* gene	This study
C50041∆*trmE*	C50041 with a defined deletion of the *trmE* gene	This study
C50041∆*trmE*::*trmE*	C50041∆*trmE* with pBR322 expressing the *trmE* gene	This study
*E.coli* χ7213	Its growth for pGMB152 with DAP, as conjugal donor	[15]
Plasmids		
pUT mini-Tn5Km2	Transposon delivery vector, Cm^r^, Km^r^	[17]
pGMB152	pGMB151 derivative, suicide vector, Amp^r^, Sm^r^, *Lac*ZYA	[15]
pBR322	For construction of SE∆*trmE*::*trmE*, Cm^r^	[18]
pBR322-*trmE*	pPR322 derivative containing *trmE*, Amp^r^ and Tet^r^	This study
Cells		
RAW264.7	Murine macrophages	This study
J774A.1	Murine macrophages	This study

**Table A2:** Primers used in this study.

Primer Name	Primer Sequences (5′-3′)	Target
*trmE*-up-F	CCCCCCCTGCAGGTCGACGTGGTTCCCGTCAGGTCT	Construction for C50041∆ *trmE*
*trmE*-up-R	CAGCCTACACAATCGCTCAAGGTTAGTCTCAACTTTGTTGCAAT	
*trmE*-Cm-F	ATTGCAACAAAGTTGAGACTAACCTTGAGCGATTGTGTAGGCTG	
*trmE*-Cm-R	TTTGTAGGCCCGGTAAGCATATGGGAATTAGCCATGGTCC	
*trmE*-down-F	GGACCATGGCTAATTCCCATATGCTTACCGGGCCTACAAA	
*trmE*-down-R	CTTATCGATACCGTCGACCAGGTAAACGGAGAAGGCGA	
*trmE*-NYZ-F	AAACTCGTTACAGGGGGCAT	
*trmE*-NYZ-R	TACGCTCAACTTCGTCGCTG	
pGMB152-F	CGTGGAGGCCATCAAACCAC	
pGMB152-R	CGCGAAATAAACGACCGGGA	
C-*trmE*-F	TTATCATCGATAAGCTTATGAGCCATAACGACACTATCGTC	Construction of complemented mutant C50041∆ *trmE*:: *trmE*
C-*trmE*-R	TCCGGCGTAGAGGATCCTTGTAGGCCCGGTAAGCATC	

Note: The underlined segments indicate the homologous sequences for the one-step ligation of recombinant plasmid.

**Table A3:** Primers used in this study.

Primer Name	Primer Sequences (5′-3′)	Size (bp)
IL-1β-F	TGGCCTTCAAAGGAAAGAATCTATACCTGTCC	167
IL-1β-R	GTTGGGGAACTCTGCAGACTCAAACTCCAC	
IL-12-F	TGCCCCCACAGAAGACGTCTTTGATGAT	138
IL-12-R	GATGGCCACCAGCATGCCCTTGTC	
TNF-α-F	CAGGCCTTCCTACCTTCAGACCTTTCCAGAT	
TNF-α-R	ACACCCCGCCCTTCCAAATAAATACATTCAT	122
IFN-γ-F	GCCAAGACTGTGATTGCGGGGTTGTATCT	
IFN-γ-R	TAAAGCGCTGGCCCGGAGTGTAGACA	198
GAPDH-F	CAGCCTCGTCCCGTAGACAA	
GAPDH-R	ACCCCGTCTCCGGAGTCCATCACAAT	156
